# Insights into nanostructured lipid carriers for the effective delivery of bioactives in swine and poultry health: review

**DOI:** 10.5713/ab.250901

**Published:** 2026-02-06

**Authors:** Noor Saba, Mohammad Moniruzzaman, Do Thi Cat Toung, Sungyeon Chin, Lin Lat La Min, Adhimoolam Karthikeyan, Dong I. Lee, Ji-Yeon Chun, Taesun Min

**Affiliations:** 1 Language Education Institute of the Office of International Affairs, Jeju National University, Jeju, Korea; 2 Department of Animal Biotechnology, Jeju International Animal Research Center (JIA) & Sustainable Agriculture Research Institute (SARI), Jeju National University, Jeju, Korea; 3 Subtropical Horticulture Research Institute, Jeju National University, Jeju, Korea; 4 Department of Biomedical Engineering, Johns Hopkins University School of Medicine, Baltimore, MD, USA; 5 Department of Food Bioengineering, Jeju National University, Jeju, Korea; 6 Food Tech Center (FTC), Jeju National University, Jeju, Korea

**Keywords:** Bioactive Compounds, Bioavailability, Health Management, Nanostructured Lipid Carriers, Poultry, Swine

## Abstract

Nanostructured lipid carriers (NLCs) are promising in target and efficient delivery of bioactive compounds with high loading capacity of bioactives, better physical stability, better encapsulation efficiency, solubility and bioavailability in comparison to the traditional delivery systems for lipophilic and hydrophobic bioactives such as essential oils and phytochemicals as well as functional feed ingredients. There are different types of NLCs combining solid lipid and liquid lipid in a single nanoparticulate matrix that have advantages over other drug delivery systems. The NLCs can be synthesized in form of imperfect, amorphous, oil-enriched, surface modified or functionalized, hybrid or composite and multiple-compartment or double-shell NLCs. Moreover, the synthesized NLCs safety, efficacy, toxicity, encapsulation efficiency, drug loading and releasing capacity, reproducibility in large scale were evaluated. The application of NLCs for the effective and target delivery of bioactive compounds are widely reported in biomedical and therapeutic studies. However, utilization of NLCs for the effective delivery of bioactive compounds is very limited in swine and poultry health. There are some recent studies reported that NLCs with can enhance growth, antioxidant capacity, immunity, gut health and microbiome as well as disease resistance in swine and poultry. This review focuses on recent developments and future prospects of utilizing NLCs in swine and poultry health management. Regardless of the potential beneficial effects of NLCs in nanodelivery of bioactive compounds, further research on long-term field oriented studies in livestock and poultry farms and economic analysis of manufactured NLCs should be conducted.

## INTRODUCTION

The intensification of swine and poultry production over recent decades has delivered dramatic gains in productivity, but it has also increased industry reliance on pharmaceutical and nutritional interventions to maintain animal health and feed efficiency. Among these interventions, antibiotic growth promoters (AGPs) historically played a dominant role in improving weight gain, feed conversion ratio and overall health. However, recognition of the public- and animal-health risks posed by antimicrobial resistance (AMR) has driven global efforts to reduce non-therapeutic antimicrobial use in livestock and to seek effective and sustainable alternatives based on One Health approach [1]. Policy responses and guidance from international organizations have encouraged reduced antibiotic usage and the development of alternative strategies for disease control and performance enhancement in food animals. These shifts have created an urgent need for novel, safe, and efficacious feed additives that maintain or improve production metrics while limiting AMR selection pressure [2,3].

Natural bioactive compounds including essential oils (EOs), plant phenolics, and phytogenics have received considerable attention as alternatives to AGPs because of their antioxidant, antimicrobial, and immunomodulatory properties [4,5]. When used appropriately, these compounds can support gut health, enhance antioxidant status, and modulate the microbiome in ways that favor growth and resilience to diseases. Nevertheless, the practical implementation of many natural additives is hampered by physicochemical and biological limitations such as low water solubility, volatility, susceptibility to oxidation or thermal degradation during feed processing, rapid clearance or poor intestinal absorption after ingestion, and inconsistent efficacy arising from batch variability [5]. These issues often necessitate higher inclusion levels, which raises cost and may negatively impact feed palatability or sensory qualities. As a result, the full potential of many promising phytogenic and nutraceutical compounds remains unrealized in commercial swine and poultry systems [4].

Nanotechnology offers tools to overcome many of the delivery challenges associated with lipophilic or labile bioactives, and lipid-based nanocarriers have emerged as particularly promising for oral administration in food animals [4–7]. Nanostructured lipid carriers (NLCs) are lipid nanoparticles (LNPs) composed of a mixture of solid and liquid lipids, which provide advantages over traditional lipid systems in terms of improved loading capacity, controlled release, and enhanced stability of incorporated bioactive compounds [6–8]. This mixed lipid architecture provides a less-ordered crystal structure than solid-only systems, enhancing drug/bioactive loading capacity and reducing expulsion of encapsulants during storage [7]. NLCs can protect labile compounds from environmental and gastrointestinal degradation, improve dispersibility in aqueous environments, enable controlled release in the gut, and enhance mucosal uptake through increased solubilization and possible transcellular absorption pathways [9]. NLCs already have shown the efficacy for nanodelivery of bioactive compounds in therapeutic applications including cancer treatments [10,11]. Importantly for feed applications, NLC components can be selected from food-grade lipids and generally recognized as safe (GRAS) surfactants, facilitating compatibility with existing feed manufacturing practices [9,12]. The combination of protection, controlled release, and improved bioavailability makes NLCs an attractive platform for delivering EOs, fat-soluble vitamins, carotenoids, and other phytochemicals using *in vitro* and *in vivo* animal models [8,13–31]. Recently, some studies have been reported the beneficial role of NLCs in delivering bioactive compounds in companion and aquatic animals especially dogs, fish and shrimp [13,14,17]. In a study, Sawatphakdee et al [13] found that NLC loaded with hydrophobic alpha-mangostin and clove oil showed greater effectiveness in inhibiting bacterial growth than the antibiotics tested, particularly against bacteria from the oral cavities of dogs. Kengkittipat et al [14] demonstrated that a natural compound, andrographolide, derived from *Andrographis paniculata* loaded NLCs enhanced bioavailability and therapeutic efficacy, supporting their potential as a functional dietary additive to promote growth and improve disease resistance in Nile tilapia (*Oreochromis niloticus*). Similarly, Yostawonkul et al [18] reported the utilization of mangosteen (*Garcinia mangostana*) peel extract loaded in nanoemulsion (MSNE)-supplemented diets in tilapia fish which could improve the growth performance, immune response, and disease resistance in tilapia fish. Furthermore, Kaewmalun et al [17] postulated that clove oil-NLC could be a potent alternative nanodelivery platform for increasing the anesthetic activity of clove oil in whiteleg shrimp (*Penaeus vannamei*). In the context of terrestrial and non-ruminant (e.g. swine and poultry), nanoformulations of EOs and other natural antimicrobials have improved the bioactivity and storage stability [4]. Moreover, there have been successful examples where NLCs have been used to enhance the oral bioavailability of pharmaceuticals in broilers (e.g. tilmicosin), demonstrating that NLCs can improve uptake and possibly reduce required dosages [8].

Translating these promising laboratory and controlled-trial findings into practical feed additives for commercial swine and poultry production requires careful attention to several interrelated issues. First, formulation and scale: NLC manufacture must be robust, reproducible, and amenable to large-scale production with consistent particle size, encapsulation efficiency, and long-term stability under feed storage conditions (temperature, humidity, mechanical stress). Second, dosage form and administration routes must align with typical feed delivery systems whether as top-dress, premix, or incorporated into pelleted feeds without degrading NLC integrity during pelleting or storage. Third, safety and toxicology: although NLCs are composed of lipids and excipients with favorable safety profiles, their nanoscale size raises specific toxicological and regulatory questions (e.g., absorption kinetics, tissue residues, and potential for unintended distribution). Fourth, efficacy endpoints should be meaningful to industry (growth performance, feed conversion ratio, mortality, morbidity, and measurable shifts in gut microbiota or immune markers) and should demonstrate consistent effects across production environments. Finally, economics and regulatory acceptance will determine adoption: NLC-based additives must be cost-competitive and acceptable to regulatory agencies that increasingly scrutinize nanomaterials in the food chain. Addressing these translational barriers will require interdisciplinary collaboration among formulation scientists, animal nutritionists, veterinary pharmacologists, and regulatory experts [32]. At the same time, NLCs present unique opportunities that align with industry trends toward precision nutrition and reduced antimicrobial reliance. By tailoring lipid composition, particle size, and surface properties, NLCs can be engineered for targeted release at specific gut sites, co-deliver complementary actives (e.g., an EO plus a vitamin or probiotic), and stabilize complex botanical extracts that otherwise show inconsistent field performance. The ability to protect oxidizable compounds (e.g., carotenoids, omega-3 fatty acids) also opens avenues for value-added functional feeds that improve meat quality and animal health. From a One-Health perspective, technologies that reduce the routine use of antibiotics while maintaining productivity would be highly desirable for both animal welfare and public-health perspectives [32].

This review synthesizes the literatures on NLCs with the aim of mapping their prospects as feed additives for swine and poultry industries. We summarize here the physicochemical principles of NLC design relevant to feed applications and the evidence for improved stability and oral bioavailability of representative actives. Later, we examine the animal studies published to date focusing on performance, health, antimicrobial, immunological, and microbiome outcomes and evaluate consistency, limitations, and gaps in the evidence. Finally, we discuss regulatory, manufacturing, and safety considerations that will influence commercial adoption, and identify priority research areas (for example, long-term safety studies, effects under commercial field conditions, cost-benefit analyses, and standardization of manufacturing and quality control). Our goal is to provide a focused, evidence-based assessment to inform researchers and industry stakeholders interested in deploying NLC technology for sustainable, antibiotic-reduced swine and poultry production.

## TYPES AND STRUCTURES OF NANOSTRUCTURED LIPID CARRIERS

**Figure 1:**
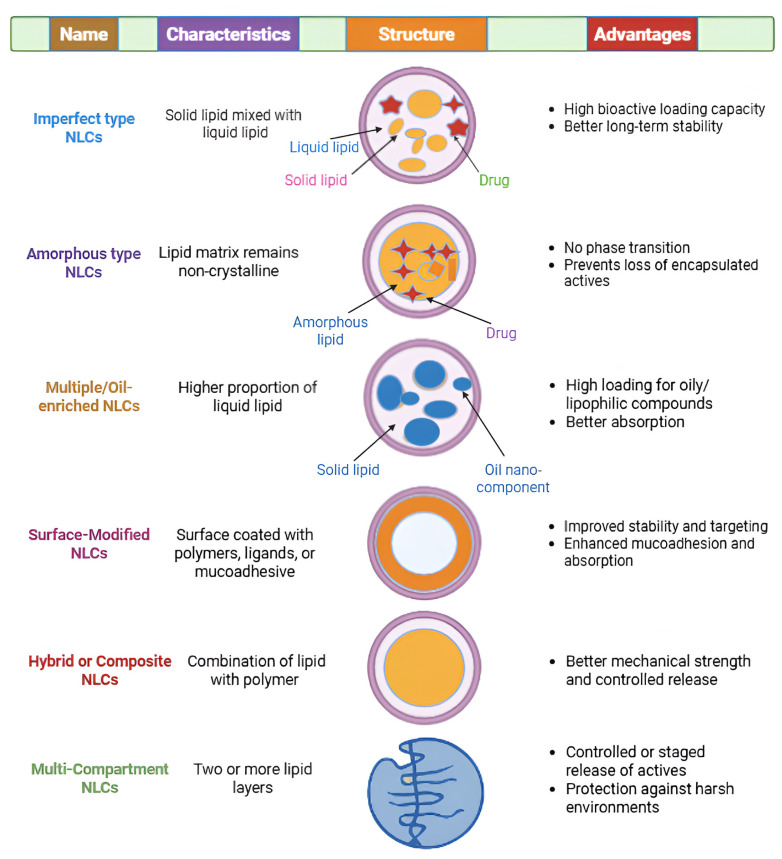
Diffrent types of nanostructured lipid carriers (NLCs) with their structures, characteristics and advantages.

NLCs are a class of lipid-based nanocarriers combining solid lipid (s) and liquid lipid (s) in a single nanoparticulate matrix. The incorporation of liquid lipid (s) into a solid lipid lattice yields a less-ordered internal structure with “imperfections,” which enhances drug (or bioactive) loading capacity, reduces expulsion during storage, and can modulate release behavior [33]. Various structural variants or types of NLCs have been proposed depending on the internal lipid configuration, nanoparticle architecture, or functional modifications. Below are some of the commonly described types of NLCs, with their distinguishing features and potential relevance to feed additive delivery (Figure 1).

### Imperfect type nanostructured lipid carriers

*Definition and structure:* The so-called *“imperfect type”* NLC is the canonical form: the solid lipid matrix is deliberately “doped” with a moderate amount of liquid lipid (oil), which disrupts the crystallinity of the solid lipid structure. The resulting lattice is imperfect (i.e. contains voids, less ordered lipid packing), allowing more “space” to accommodate the bioactive molecules (or actives) and reducing the tendency of the matrix to expel the cargo during aging. This is the most frequently reported form of NLCs [33].

*Advantages and considerations:* Because the crystalline lattice is partially disrupted, higher loading and better retention over time are possible. However, if too much liquid lipid is used, there is risk of phase separation or that the matrix becomes too “soft,” losing structural integrity.

### Amorphous type nanostructured lipid carriers

*Definition and structure:* In an *“amorphous type”* NLC, the idea is to maintain the lipid matrix in a non-crystalline (amorphous) state altogether. This is typically achieved by blending specific lipids that, when combined, resist crystallization and instead form an amorphous (glassy) lipid phase. The lack of crystallinity ideally eliminates phase transitions and expulsion of cargo during storage [32].

*Advantages and considerations:* Because no crystallinity is present, the risks associated with polymorphic transitions (which might push out the active) are minimized. But creating and maintaining a stable amorphous matrix can be challenging, especially under temperature fluctuations or during feed processing (pelleting, heat).

### Multiple/oil-enriched/“ultra-small” or high-oil nanostructured lipid carriers

*Definition and structure:* In these variants, a relatively higher proportion of liquid lipid is incorporated into the NLC, yielding what is sometimes called an *oil-enriched NLC* or *ultra-small NLC*. The term “ultra-small” generally refers to particle size, but in many reports these formulations are enriched in oil relative to conventional NLCs to further enhance payload and flexibility of internal structure. For example, “oil-enriched ultra-small NLCs (usNLCs)” have been proposed, wherein the liquid lipid is more pronounced within the nanoparticle core [34].

*Advantages and considerations:* The greater oil content can increase loading of highly lipophilic actives. However, too high oil content may compromise the structural stability (leading to leakage or phase separation). Also, small sizes may enhance absorption but may also raise handling or stability challenges.

### Surface-modified or functionalized nanostructured lipid carriers

Beyond the internal lipid architecture, NLCs can be modified at their surface to tailor interaction with the biological milieu (e.g. gut mucosa) or to provide targeting, improved mucoadhesion, or co-delivery. While not strictly a “type” of NLC in internal structure, surface modifications are functionally a major variant class:

*PEGylated nanostructured lipid carriers:* surface modified with polyethylene glycol (PEG) to improve colloidal stability, reduce opsonization, or reduce aggregation in biological fluids (general nanoparticle strategy).

*Ligand-targeted nanostructured lipid carriers:* decorating the surface with ligands (e.g. lectins, peptides) to target specific intestinal cell receptors or for site-specific adhesion.

*Mucoadhesive-modified nanostructured lipid carriers:* using chitosan, alginate, or other mucoadhesive polymers on the surface to prolong residence time in the gastrointestinal tract, potentially increasing local absorption of actives.

Some studies in pharmaceutical NLC design incorporate such strategies to improve stability, targeting, or absorption [35].

### Hybrid or composite nanostructured lipid carriers (lipid–polymer, lipid–inorganic)

Some NLC designs integrate additional structural elements (polymers or inorganic components) to gain hybrid properties:

*Lipid–polymer hybrid nanostructured lipid carriers:* embedding or coating the lipid core with a polymeric shell (or integrating polymer chains) to combine the advantages of lipids (biocompatibility, ease of release) and polymers (mechanical stability, controlled permeability).

*Lipid–inorganic hybrid nanostructured lipid carriers:* incorporating inorganic nanoparticles (e.g. silica, gold, iron oxide) within or on the surface of NLCs to add functionalities (e.g. imaging, magnetic responsiveness, triggered release).

Though less common in feed or oral delivery literature, such hybrids are explored in drug delivery settings and might be relevant in the future for “smart” or multi-functional feed additives. General reviews of LNP systems mention hybrid strategies as future directions [36].

### Multi-compartment/double-shell or core–shell nanostructured lipid carriers

Another variant is core–shell or “double-shell” NLCs, where a secondary lipid shell or barrier is built around the core, or multiple compartments (e.g. inner and outer lipid shells) are engineered. This architecture may allow staged release or improved protection of sensitive actives. Although less commonly described under the “NLC” name in oral delivery literature, the concept is akin to layering strategies used in LNP design to control release kinetics or protect cargo under harsh gastrointestinal environments [37].

## APPLICATION OF NANOSTRUCTURED LIPID CARRIERs IN SWINE HEALTH

NLCs have been explored as a strategy to improve the oral pharmacokinetics and bioavailability of veterinary antibiotics in pigs. For example, tilmicosin (TMS) is widely used to treat bacterial infections in verterinary medicine, however due to its poor solubility, bitterness, gastric instability and intestinal transport barrier make it limited in use. The NLC using hot emulsion with stearic to oleic acids found to be an effective oral delivery carrier for overcoming obstacle of TMS on oral adninistration [38]. Wen et al [38] optimized tilmicosin-loaded NLCs (TMS-NLCs) and reported improved physicochemical stability and sustained-release characteristics; *in vivo* absorption studies in animal models indicated that NLC encapsulation can enhance oral exposure compared with conventional formulations, suggesting potential to reduce dosing or improve therapeutic windows in swine infections. Furthermore, the TMS-NLCs showed the same antibacterial effect as free TMS. This type of formulation work is directly relevant for swine respiratory and systemic infections where oral medication is preferred [38].

Beyond antibiotics, reviews addressing encapsulation strategies for zootechnical additives summarize approaches applicable to swine nutrition including NLCs for EOs, organic acids, and other phytogenics and discuss how encapsulation improves stability, targeted release, and palatability in feed matrices. Fontana et al [39] provide an overview of methods and examples for poultry and swine, highlighting that properly designed lipid carriers can protect actives during pelleting and release them in the intestine where they exert their effects. EOs and plant extracts are attractive AGP alternatives as they have strong antimicrobial, anti-inflammatory, antiviral, antioxidant and immunomodulatory properties in pig production, but their volatility and poor aqueous dispersibility limit efficacy. Furthermore, Stevanović et al [40] discuss how nanoencapsulation particularly in lipid-based systems like NLCs mitigates these limitations by protecting volatile compounds from oxidation and evaporation, improving dispersion in water/feed, and enabling controlled release in the gut; these properties support use of EO-NLCs to modulate gut microbiota, antioxidant status, and growth in piglets and growing pigs. Studies focused on nano-encapsulation in agri-food systems emphasize that nanoparticle carriers (including NLCs) can improve the stability and intestinal delivery of organic acids and EOs used as zootechnical additives in swine diets. Mahato et al [41] review nanoencapsulation strategies across agri-food applications and note specific examples where encapsulated phytogenics or acids demonstrate better shelf life, controlled release, and preserved bioactivity all desirable traits for pig feed additives that must withstand storage and pelleting. Experimental studies of EO-based nanocarriers demonstrate antimicrobial activity against foodborne or enteric pathogens relevant to pigs. Ojeda-Piedra et al [42] reviewed nano-encapsulated EOs as preservation agents and documented numerous cases where lipid nanocarriers improved antimicrobial potency and reduced volatility. Translating these findings into swine, EO-NLCs could be used to lower enteric pathogen carriage (e.g., *Salmonella*, *Escherichia coli*) or reduce post-weaning diarrhoea by delivering actives to the gut mucosa more effectively than free oils [42]. Some studies examined surfactant and lipid choices that protect labile actives from gastrointestinal enzymes highly relevant for swine where digestive enzyme activity can degrade feed additives. Shahzadi et al [43] evaluated NLC surfactant types for enzymatic protection of incorporated peptides and showed that surfactant identity strongly influences enzymatic stability and release. For swine applications (e.g., delivery of bioactive peptides, enzymes, or probiotics), these formulation principles indicate how NLC design can be tuned to survive the gastric/intestinal environment and release payloads where needed.

Review studies comparing solid lipid nanoparticles (SLNs) and NLCs discuss why NLCs are preferable for many feed-additive applications in livestock due to higher loading capacity and reduced cargo expulsion during storage characteristics that matter for swine feeds stored in variable farm conditions [44,45]. Viegas et al [44] systematically compared SLNs and NLCs and highlighted how NLC formulation choices (lipid blend, liquid lipid fraction) influence long-term stability and release practical considerations for designing pig feed additives that must remain stable through storage and feed processing. Cross-species application studies (e.g., aquaculture and poultry) illustrate biological outcomes that are informative for swine: nano-encapsulated phytochemicals improved growth, feed conversion, and disease resistance in fish and birds under challenge conditions. The NLC synthesizing outcomes and point to analogous opportunities in pigs, such as enhancing post-weaning resilience, improving nutrient utilization, and reducing reliance on antibiotics. These cross-species insights help prioritize which bioactives and NLC designs to test first in swine. A lipid-encapsulated zinc oxide (ZO) supplement was tested in weanling pigs and demonstrated improved growth performance, intestinal morphology, and digestive enzyme activities compared with non-encapsulated ZO. For instance, in a study by Jang et al [46], the use of lipid-encapsulated ZnO containing 8%, 10% or 12% lipid improved villus height and enzyme activity in the small intestine of piglets, indicating better nutrient absorption and gut development during a critical post-weaning period. This suggests that lipid-based nanoparticulate or microencapsulated mineral formulations can effectively enhance gut health and performance in swine. Moreover, nano-encapsulation of n-3 polyunsaturated fatty acids (PUFAs) such as docosahexaenoic acid (DHA) sourced from fish oil or algal oil delivered to gestating gilts and their piglets improved antioxidant enzyme activity and DNA repair capacity in the livers of newborn piglets, compared with conventional oil forms [47]. Though not strictly an NLC study, the work by Kowalczyk et al [47] show how nanoscale delivery systems in swine can impact neonatal health outcomes, particularly through improved bioavailability and downstream physiological effects. It highlights the potential for nanocarrier systems in early-life programming of piglets via maternal supplementation. Nano chitosan–zinc complexes were fed to weaned piglets, and the piglets showed improved growth performance and antioxidant capacity of the small intestine compared to controls. Although the formulation is not labelled as “NLC,” it demonstrates how nanoscale delivery of minerals in piglet diets can enhance gut health and performance -a model that could be translated into NLC-based mineral or bioactive feed systems in swine [48].

Microencapsulated EOs containing stearic acid, along with a blend of EOs (20% cinnamaldehyde, 2% thymol and 3% carvacrol) were provided in the diet of weaned piglets and resulted in enhanced growth performance, improved intestinal structure, and reduced diarrhea incidence compared to non-encapsulated oils. While this study used microencapsulation rather than NLCs, it illustrates the utility of encapsulation technology in piglet nutrition, reinforcing the promise of NLC systems for analogous applications [49]. Ivermectin as antiparasitic agent encapsulated in amorphous NLCs (diameter of 153.5 nm) with a zeta potential of −31.5 mV, 95.72% encapsulation efficiency and 11.17% drug loading capacity demonstrated markedly improved intracellular delivery and antiviral potency against porcine epidemic diarrhea virus (PEDV) in cell models [50]. Xu et al [50] reported high encapsulation with viral load reductions of up to three orders of magnitude versus free ivermectin evidenced that NLC-formulation can enhance the antiviral efficacy of repurposed drugs relevant to swine viral disease control.

Lipid-encapsulated ZnO (micro/nano-scale lipid delivery) fed to weanling pigs improved intestinal morphology (villus height), digestive enzyme activity, and growth performance compared to the unencapsulated ZnO [46]. This demonstrates that lipid encapsulation of inorganic feed additives can protect actives through the stomach and improve intestinal uptake, a translational principle directly applicable to designing mineral- or phytogenic-loaded NLCs for piglets. LNP platforms (closely related to NLC technology) have been successfully used in pigs for intramuscular DNA/mRNA delivery and vaccination (e.g., single-dose intramuscular LNP-DNA gave robust systemic and mucosal responses in pigs). These vaccine studies highlight the feasibility and immunogenic potential of lipid carriers in porcine species and suggest NLC-based adjuvants or oral vaccine carriers could be developed for swine disease control [51].

Recent reviews and experimental studies on EO nano-formulations show improved antimicrobial potency, reduced volatility, and targeted release desirable for controlling enteric pathogens in swine (e.g., *Salmonella*, *E. coli*). Ojeda-Piedra et al [42] summarized many EO nanoencapsulation examples with improved antimicrobial and stability profiles that support testing EO-NLCs in pig diets to reduce post-weaning diarrhea and pathogen carriage. *In vitro* studies using natural-lipid nanocarriers reported enhanced interaction with bacterial membranes and improved antimicrobial potency against poultry/foodborne pathogens (mechanisms applicable to pig enteric pathogens). Ribeiro et al [52] provided mechanistic data that supports translating lipid-carrier approaches to target enteric bacteria in swine. Comprehensive reviews on NLCs for oral delivery synthesize key parameters (particle size, lipid blend, liquid lipid fraction, surfactant choice, drying/scale-up methods) that determine success for oral administration [37,53]. These reviews discuss case studies relevant to veterinary oral dosing and are practical guides for designing swine-compatible NLC feed additives. They also flag regulatory and scale-up considerations specific to food-animal use [37]. Recent works on formulated cell-free supernatants (from beneficial microbes) into NLCs found potent antibacterial activity and improved stability which are intriguing concepts for swine where probiotic metabolites could be delivered as stabilized antimicrobial NLC additives to reduce pathogen pressure without antibiotics. These hybrid bioactive-NLC approaches open novel avenues for gut health management in pigs (Table 1) [53].

## APPLICATIONS OF NANOSTRUCTURED LIPID CARRIERs IN POULTRY HEALTH

NLCs have been utilized to enhance the oral administration and pharmacokinetics of veterinary antibiotics in broiler chickens. A notable example includes the formulation of tilmicosin-loaded NLCs, which exhibited better gastrointestinal stability and improved cellular permeability compared to traditional formulations. In broilers, the optimized NLCs led to increased relative oral bioavailability and showcased sustained release properties, potentially allowing for reduced dosing or better therapeutic windows for respiratory and systemic infections. This research highlights the straightforward translational potential of NLCs to enhance drug exposure following oral administration in poultry [8]. Essential-oil-based NLCs have been created and evaluated specifically for controlling pathogens in poultry products. Pires et al [54] formulated NLCs containing a variety of EOs (e.g., cinnamon, lemongrass, clove, oregano) and observed strong antimicrobial effects against *Campylobacter* spp. isolated from chicken carcasses; notably, several EO-NLCs maintained physicochemical stability for long-term storage (months) at room temperature, which underscores their applicability for food-chain uses (e.g., carcass decontamination or as feed/top-dress additives to limit colonization). The NLCs demonstrated strong antimicrobial properties at low concentrations (approximately 0.2 mg/mL), preserved physicochemical stability for 210 days, and showed acceptable safety in chicken embryo tests, indicating their potential for lowering pathogen levels in poultry farming and enhancing food safety. This inquiry illustrates how NLCs can stabilize volatile phytochemicals and enhance their antimicrobial efficacy in poultry settings [54].

An extensive review focusing on EOs and their nanoformulations for poultry feeding summarizes various applications where lipid-based nanosystems (such as NLCs and nanoemulsions) tackle the main drawbacks of free EOs in terms of volatility, oxidation, low aqueous dispersibility, and inconsistent gut delivery. The review compiles evidence showing that nanoformulations can boost antioxidant status, modulate gut microbiota, and improve growth-related results in broilers when compared to non-encapsulated EOs, while also identifying gaps like the necessity for large pen/field trials and evaluations of pelleting stability. The reason of enhanced growth, antioxidant status and gut microbial activities in broilers may be due to the presence of chemical agents in EOs such as terpenes (α-pinene, ar-curcumene and limonene), terpenoids (menthol, linalool, thymol and camphor) and phenylpropanoids (eugenol and cinnamaldehyde) [2]. The mechanism of NLCs effectiveness can be explained by its ability to solubilize the hydrophobic and lipophilic EOs as well as slow releasing and target delivery of EOs with their high absorption and utilization rate in the intestine of animals. This article offers a thorough background connecting EO nano-delivery directly to outcomes related to poultry health mangement [2].

Research examples from pharmacological points of view indicate that NLCs can safeguard delicate botanical oils from deterioration and deliver them effectively in biological systems. A study involving Amazonian flora, red sacaca (a plant EO from *Croton cajucara*) incorporated into NLCs documented enhanced physicochemical stability, controlled release, and preserved bioactivity in vitro that are directly applicable to poultry feed scenarios where feed processing or gut conditions might otherwise deactivate the oils. Such formulation research demonstrates practical techniques (selecting lipids, surfactant choices, production methods) that can be modified to produce effective feed-grade NLC additives as the health-promoting phytochemicals [55].

A recent review on LNPs carrying EOs compiles evidence from human, veterinary, and food-industry uses, emphasizing how LNPs and NLCs improve antimicrobial effectiveness, reduce volatility, and allow for controlled release. The review stresses practical considerations for poultry applications including the choice of food-grade lipids and surfactants, stability during long-term storage, and regulatory factors and calls for additional *in vivo* poultry trials examining growth performance, immune markers, and microbiome alterations. This review article serves as a helpful guide for researchers aiming to transition of NLC-EO formulations from laboratory settings to practical applications in poultry production [56].

Recent research conducted by Zhang et al [57] created maduramicin ammonium-loaded nanostructured lipid carriers (MAD-NLCs) to improve anticoccidial effectiveness in broiler chickens. They fine-tuned the NLC formulation using a Box–Behnken design, resulting in a particle size of approximately 153.6 nm, a zeta potential of −41.4 mV, and a high encapsulation efficiency of around 90.5%. In *in vivo* experiments, these MAD-NLCs exhibited significantly greater control over *Eimeria tenella* compared to the unencapsulated drug, with lower toxicity and sustained release characteristics, indicating that NLC delivery might facilitate the use of lower doses and enhance safety [57].

**Figure 2:**
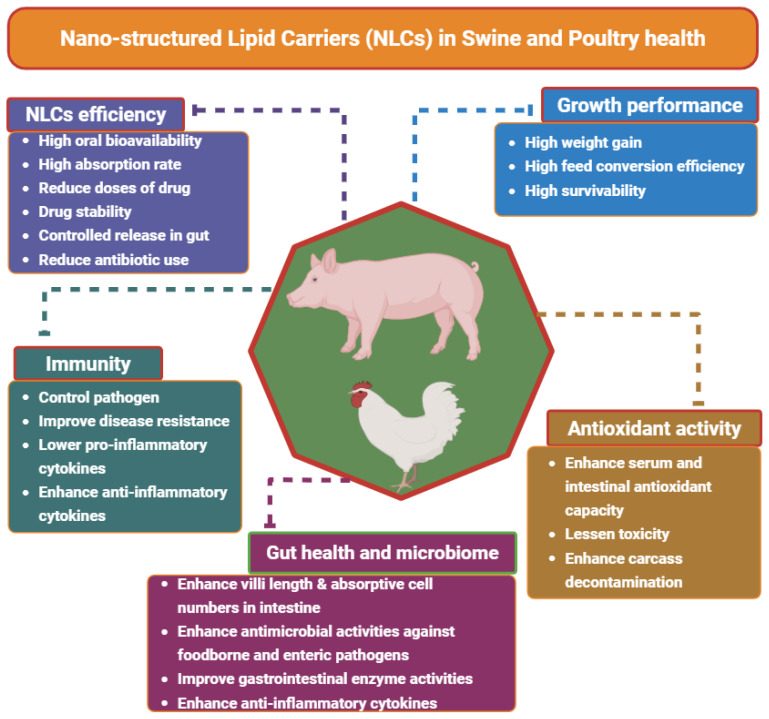
Schematic diagram on effects of nanostructured lipid carriers on swine and poultry health.

Ribeiro et al [52] explored nanocarriers composed of natural lipids to combat *Campylobacter jejuni in vitro*. Although their lipid nanocarriers did not involve comprehensive *in vivo* feeding studies, they still illustrate how NLC-like systems can interfere with bacterial cell functions, improve the stability of antimicrobial lipids, and lessen toxicity. Despite being an *in vitro* study, it provides significant mechanistic evidence that such nanoparticle systems can be both safe and effective prior to trials in live birds (Table 2, Figure 2) [52].

## CHALLENGES AND FUTURE OUTLOOK OF UTILIZING NANOSTRUCTURED LIPID CARRIERs

One of the foremost practical challenges for translating NLCs into livestock feeds is adapting laboratory production methods to industrial scales while preserving particle uniformity and encapsulation performance. Small-scale techniques commonly used in academia (ultrasonication, batch high-shear homogenization) can yield high-quality NLCs in the lab but are often difficult to reproduce reliably at tonnage scale; Khairnar et al [58] review scale-up strategies including high-pressure homogenization, microfluidization, and spray-drying approaches and highlight engineering, process-control, and equipment-validation steps that will be needed to make NLC manufacture robust and cost-effective for feed applications.

Closely linked to scale-up is the need for quality-by-design (QbD) frameworks and rigorous process analytical technology (PAT) to assure consistent NLC quality over large production runs. Hidayat et al [59] describe QbD-driven NLC optimization identifying critical material attributes (lipid type, liquid-lipid fraction, surfactant), critical process parameters (temperature, shear, cooling rate), and control strategies and recommend in-process monitoring (particle size, polydispersity, zeta potential) to prevent drift in product attributes during scale-up, a strategy directly applicable to feed-grade NLC development.

Long-term physicochemical stability of NLCs under real-world storage and feed-processing conditions is another bottleneck. Mehrdadi [60] discuss how lipid recrystallization, polymorphic transitions, and oxidative degradation of lipid matrices and payloads can lead to drug expulsion, aggregation, and loss of functionality; they emphasize formulation measures (antioxidants, lipid selection, optimized liquid-lipid fraction) and stabilization strategies (drying, lyophilization, protective carriers) that need to be tailored to the thermal and mechanical stresses of feed manufacture and farm storage.

Regulatory uncertainty and harmonized risk-assessment pathways pose a major non-technical challenge for adoption of NLCs in the feed chain. The European Food Safety Authority (EFSA) Scientific Committee [61] guidance outlines data expectations for nanomaterials in food and feed including characterization, toxicokinetics, residue studies, and specific nanotoxicology endpoints and signals that even GRAS components require nanoparticle-specific evaluation; aligning NLC development with these regulatory requirements early will be essential for market acceptance.

Environmental fate and ecotoxicology of nano-lipid materials excreted in manure or entering soils is an under-studied but important concern. Muzammil et al [62] review nanomaterial toxicity in agricultural systems and stress the need for studies on persistence, microbial community shifts, and trophic transfer of nanoparticles from manure to soil and water; such environmental risk assessments should accompany any large-scale deployment of NLC-based feed additives.

Robust analytical and characterization methods are required to define NLC critical quality attributes and to track them in complex feed matrices and biological samples. Abbasi et al [63] summarize how particle size, surface charge, and coating chemistry influence nanoparticle–biological interactions and recommend standardized methods such as dynamic light scattering (DLS), transmission electron microscope (TEM)/scanning electron microscope (SEM), chromatographic assays for payload, mass-balance/residue analysis that regulators and researchers can use to compare formulations and to detect residues in edible tissues.

Economic feasibility remains a decisive determinant of whether NLCs will be implemented in mainstream livestock nutrition. Dhiman et al [64] analyze cost drivers for LNP production (raw lipid costs, surfactants, energy for homogenization/drying, and downstream formulation steps) and argue that cost reductions will require process intensification, use of inexpensive food-grade lipids, and integration of NLC production into existing feed-mill workflows to avoid prohibitive per-ton costs.

Socio-regulatory acceptance and consumer perception will influence market uptake; transparent labeling, risk communication, and stakeholder engagement are needed. Schoonjans et al [65] outline European approaches to nanoparticle safety assessment and underscore the importance of clear regulatory frameworks and public communication to build consumer trust for nanomaterials in the food chain lessons that feed-additive developers must heed if NLCs are to gain broad acceptance.

Risk-management strategies for NLCs should integrate One-Health thinking, balancing animal health gains with human and environmental safety [66,67]. Paramo et al [66] emphasize interdisciplinary risk assessments that include ecotoxicology, occupational exposure for feed-mill workers, and food-chain residue monitoring; implementing such holistic frameworks will help to identify acceptable operational boundaries and mitigation measures before large-scale deployment.

Finally, the future outlook for NLCs is promising if research pivots from formulation proof-of-concept to translational studies that address the above gaps. He and Hwang [67] discuss how demonstrating real-world benefits (cost-benefit analyses, pen- and farm-level trials, and evidence of reduced antibiotic dependence) combined with standardized characterization and regulatory alignment will accelerate adoption; they also highlight opportunities in co-delivery (nutrient+ probiotic), targeted gut-segment release, and integration with precision-feeding platforms.

## CONCLUSION

In summary, the advent of NLCs offers a compelling route to address longstanding challenges in feed additive delivery for swine and poultry production. As an updated review on NLCs by Nguyen et al [68] underscores, the unique lipid matrix architecture of NLCs enables superior loading of lipophilic and volatile compounds, improved stability against environmental and gastrointestinal stressors, and enhanced oral bioavailability. As mentioned earlier, NLCs with different structures such as amorphous, imperfect, oil-enriched, surface-modified, composite or mult-compartment has different characteristics and functions in nature. This structural advantage lays the foundation for NLCs to become transformative in animal nutrition applications. The NLCs as described before, may work as nanodelivery system for the effective and target delivery of hydrophobic and lipophilic EOs and phytochemicals. The beneficial effects of synthesized NLCs rely on the solubility, stability, encapsulation efficiency, loading and releasing capacity of bioactive compounds. Moreover, for the broader implications of NLCs in veterinary nutrition and disease management are highlighted in the recent synthesis of revolutionizing veterinary medicine. The role of NLCs in advancing animal health, nutrition and disease management documented improvements in mineral and vitamin bioavailability, immune response and growth performance across farm animal species. This supports the potential of NLCs not just as the carrier of feed additives, but as integrated solutions that align with One Health goals by reducing antibiotic reliance and improving animal welfare [69]. However, meaningful translation from laboratory successes to commercial application in swine and poultry systems remains constrained by key gaps. The study developed and characterization of NLCs containing food-grade interesterified lipid phase for food application shows that for feed-grade systems, lipid composition, polymorphic behaviour, and long-term storage stability are critical and these factors may be significantly influenced by processing conditions. For feed mills operating under varied on-farm conditions, achieving reproducible NLCs batches and predictable performance remains a major practical hurdle [70]. Therefore, we need careful consideration for newly developed drug delivery system with NLCs in terms of their safety, efficacy, toxicity, reproducibility with consistent particle size, encapsulation efficiency, and long-term stability under feed storage conditions. Nonetheless, efforts should be taken considering economic significance of the newly produced drug with NLCs in swine and poultry health management.

Looking ahead, the future of NLCs in animal nutrition is promising if research and development emphasize scalable manufacturing, regulatory clarity, and integration with feed processing workflows. The application of natural bioactive compounds in animal nutrition calls for interdisciplinary collaborations among nutritionists, nanotechnologists and industry stakeholders to move from proof-of-concept to field-ready products. With concerted efforts, NLC-based feed additives could become an integral component of next-generation livestock nutrition, helping to enhance productivity, health, and sustainability across swine and poultry sectors.

## Figures and Tables

**Table 1:** Applications of nanosturctured lipid carriers (NLCs) in swine health

Type of NLCs formulation	Experimental model/target	Main results/key findings	Nutritional or health implication	References
Tilmicosin-loaded NLC (TMS-NLC)	Porcine oral model	Improved physicochemical stability and sustained release; enhanced oral exposure and bioavailability vs free tilmicosin	Enables lower antibiotic dose and improved efficacy in swine respiratory therapy	[38]
NLCs for zootechnical additives	Swine and poultry nutrition (review)	Encapsulation improved feed-additive stability, palatability, and intestinal release	Provides formulation guidance for applying NLCs in swine feeds	[39]
Essential-oil/plant-extract NLCs	Weaned piglets and growing pigs (review)	Encapsulation prevented oxidation & volatility, improved aqueous dispersion and gut delivery	Supports EO-NLCs to enhance microbiota balance and growth rate	[40]
Encapsulated organic acids/EOs	Swine feed additives	Nanoencapsulation extended shelf-life and controlled release of bioactives during pelleting	Demonstrates practicality of NLCs for stable feed formulations	[41]
Essential-oil lipid nanocarriers	Enteric pathogens (*Salmonella, E. coli*)	Increased antimicrobial potency and reduced volatility of EOs	Alternative to AGPs for pathogen control and gut health improvement	[42]
NLCs with different surfactants	Simulated GI enzyme system	Surfactant type influenced enzymatic protection and release behavior	Informs design of NLCs that survive gastric conditions in swine	[43]
Comparison of SLN vs NLC	Feed formulation stability (review)	NLCs offered higher loading capacity and less cargo expulsion during storage	Preferred carrier for long-term stable swine feed additives	[44]
Cross-species NLC	Comparative studies informing swine	NLC-encapsulated phytochemicals enhanced growth and disease resistance in animals	Identifies bioactives and NLC designs for testing in pigs	[45]
Lipid-encapsulated ZnO	Weanling piglets	Improved villus height, enzyme activity, and growth vs free ZnO	Enhances nutrient absorption and gut development post-weaning	[46]
Nano-PUFA delivery system	Gestating gilts to piglets	Increased antioxidant enzymes and DNA repair in newborn liver vs conventional oil	Improves neonatal health through maternal nanonutrient supplementation	[47]
Nano chitosan–zinc complex	Weaned piglets	Elevated growth rate and intestinal antioxidant capacity vs controls	Demonstrates benefit of nanoscale mineral delivery systems	[48]
Microencapsulated EOs	Weaned piglets	Enhanced growth, improved intestinal structure, and reduced diarrhea vs free EO	Supports encapsulation technologies for gut health and feed efficiency	[49]
Ivermectin-loaded NLC (IVM-NLC)	*In vitro* (porcine epidemic diarrhea virus, PEDV cell model)	High encapsulation (~150 nm); ~3-log viral load reduction vs free drug	Demonstrates antiviral potential of NLC-formulated agents for swine diseases	[50]
Lipid-encapsulated ZnO (repeated validation)	Weanling piglets	Replicated villus height and enzyme activity improvements vs uncoated ZnO	Confirms benefit of lipid coating for mineral bioavailability	[46]
LNP-DNA vaccine (analogous to NLC)	Pigs (intramuscular vaccination)	Single dose induced strong systemic and mucosal immune responses	Demonstrates feasibility of lipid nanocarriers for veterinary vaccines	[51]
EO nano-formulAtions (review)	Swine gut pathogens	Reported improved stAbility and controlled release of bioactives	Supports EO-NLC dEvelopment to reduce enteric pathogens	[42]
Natural-lipid nanocarriers	*In vitro* (bacterial membrane interaction)	Demonstrated enhanced antimicrobial activity and membrane disruption	Mechanistic support for lipid nanocarriers targeting pig gut pathogens	[52]
Oral NLC delivery systems (review)	Veterinary drug delivery case studies	Summarized critical parameters for oral NLC design and scale-up	Framework for developing swine-specific oral NLC feed additives	[37]
Cell-free supernatant (CFS)-loaded NLC	*In vitro* antimicrobial assay	Encapsulation improved stability and antibacterial activity of postbiotics	Novel bio-NLC approach for antibiotic-free pathogen control in pigs	[53]

EO, essential oil; AGP, antibiotic growth promoter; SLN, solid lipid nanoparticle; PUFAs, polyunsaturated fatty acids; PEDV, porcine epidemic diarrhea virus; LNP, lipid nanoparticle.

**Table 2:** Applications of nanostructured lipid carriers (NLCs) in poultry health

Type of NLC formulation	Experimental model/target	Main results/key findings	Nutritional or health implication	References
Tilmicosin-loaded NLC (TMS-NLC)	Broiler chickens/oral administration	Improved gastrointestinal stability and cellular permeability; higher relative oral bioavailability and sustained drug release vs commercial formulation	Enhances oral antibiotic exposure, allowing reduced dosage and improved therapeutic efficiency in broilers	[8]
Essential-oil-based NLCs (cinnamon, lemongrass, clove, oregano)	Chicken carcass isolates (Campylobacter spp.)/ *in vitro* and storage studies	Demonstrated strong antimicrobial activity; long-term physicochemical stability under ambient storage	Stabilizes volatile essential oils (EOs) and enhances antimicrobial efficacy for poultry feed and carcass decontamination	[54]
EO nanoformulations (NLCs, nanoemulsions)	Broiler feeding trials (review)	Summarized improved antioxidant status, microbiota modulation, and growth performance vs non-encapsulated EOs	Establishes evidence base for EO-NLCs as health-promoting feed additives; highlights need for pen-scale validation	[2]
Red sacaca essential-oil NLC	*In vitro* (formulation and bioactivity testing)	Achieved enhanced physicochemical stability, controlled release, and preserved bioactivity under simulated conditions	Provides formulation guidance for stable feed-grade NLCs protecting sensitive plant oils in poultry diets	[55]
Lipid nanoparticle and NLC systems carrying EOs	Human, veterinary, and food-industry applications (review)	Reported enhanced antimicrobial potency, reduced volatility, and controlled release of EO payloads; emphasized regulatory and storage considerations	Framework for scaling EO-NLC technology to poultry industry; supports safety and stability optimization	[56]
Maduramicin ammonium-loaded NLC (MAD-NLC)	Broiler chickens/anticoccidial trial	Particle size≈153 nm; zeta −41.4 mV; EE≈90%; significantly better Eimeria tenella control and lower toxicity than free drug	Enables lower-dose, safer anticoccidial therapy with sustained release benefits	[57]
EO-NLCs (geranium, oregano, clove)	Chicken carcass Campylobacter isolates and embryo safety assay	MIC≈0.2 mg/mL; maintained stability 210 days; no embryotoxic effects	Promising for reducing Campylobacter colonization and enhancing food safety in poultry production	[54]
Natural-lipid nanocarriers (similar to NLCs)	*In vitro* (*Campylobacter jejuni*)	Enhanced antimicrobial activity and lipid stability; reduced toxicity in cell assays	Provides mechanistic evidence supporting safe, effective lipid nanocarriers for poultry gut pathogen control	[52]

## Data Availability

Upon reasonable request, the datasets of this study can be available from the corresponding author.
